# *Panax notoginseng* Saponins Ameliorate High‐Fat Diet‐Induced Liver Injury via Mechanisms Involving TLR4‐Mediated Signaling and Lipid Metabolism

**DOI:** 10.1002/fsn3.71544

**Published:** 2026-02-13

**Authors:** Rong Li, Junyu Ma, Mengyao Li, Bangzhao Zeng, Xuexun Li, Xiaoyan Bi, Xin Zhao, Qin Gao, Yanling Yao, Yang Jiang, Chunmei Zhang, Fuli Ya

**Affiliations:** ^1^ Department of Nutrition, School of Public Health Dali University Dali Yunnan China; ^2^ School of Public Health Jining Medical University Jining Shandong China; ^3^ Department of Nutrition, The Eighth Affiliated Hospital Sun Yat‐Sen University Shenzhen Guangdong China; ^4^ Department of Laboratory Teaching Center, School of Public Health Dali University Dali Yunnan China

**Keywords:** lipidomics, liver injury, MAPK, NF‐κB, *Panax notoginseng* saponins, TLR4

## Abstract

*Panax notoginseng* saponins (PNS), the primary bioactive constituents derived from the traditional herb *Panax notoginseng*, have been used in nutritional supplements. However, their molecular mechanisms against hyperlipidemia‐induced liver injury remain to be fully elucidated. In this study, supplementation with PNS (200 mg/kg diet) for 12 weeks significantly ameliorated high‐fat diet (HFD)‐induced liver injury in C57BL/6J mice, as evidenced by improved serum lipid profiles and attenuated hepatic oxidative stress and inflammation. Lipidomics analysis indicated that PNS administration restored hepatic lipid homeostasis, particularly by normalizing dysregulated triglycerides. Mechanistically, PNS markedly suppressed the activation of key inflammatory signaling pathways, including both the TLR4/MyD88‐mediated NF‐κB and MAPK (ERK, JNK, p38) cascades. Complementary in vitro experiments in LPS‐stimulated HepG2 cells confirmed that PNS attenuated cellular injury and downregulated these pathways. Notably, the protective effect of PNS was not additive to that of the specific TLR4 inhibitor TAK‐242, indicating a functional convergence on the TLR4 signaling axis. Collectively, these findings demonstrate that PNS protects against HFD‐induced liver injury by improving lipid metabolism and attenuating hepatic inflammation via mechanisms involving the suppression of TLR4‐mediated NF‐κB and MAPK signaling, providing a refined mechanistic basis for its application as a dietary supplement in metabolic liver health.

## Graphical abstract

*Panax notoginseng* saponins (PNS) ameliorate high‐fat diet‐induced liver injury by modulating the LPS‐TLR4/MyD88 axis. This leads to the concurrent suppression of NF‐κB and MAPK signaling, which reduces oxidative stress and inflammation while improving lipid metabolism.

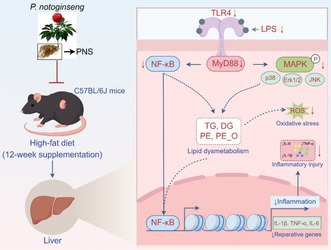

## 1 Introduction

Non‐alcoholic fatty liver disease (NAFLD), recently redefined as metabolic dysfunction‐associated steatotic liver disease (MASLD), has emerged as a pervasive global public health challenge with escalating prevalence (Hagstrom et al. 2024). Epidemiological studies indicate that approximately 38% of the global population and 7%–14% of children and adolescents are affected by MASLD. Projections suggest that the adult prevalence of MASLD will exceed 55% by 2040 (Younossi et al. 2025). In Chinese pediatric populations, MASLD prevalence has ranged from 4.4% to 7.0% (Liu, Ling, et al. 2024). As a leading etiology of chronic liver disease, MASLD substantially elevates the risk of cardiovascular events, cirrhosis, and hepatocellular carcinoma (Zhang, Li, et al. 2025). Chronic overnutrition represents a central pathogenic driver of MASLD, with high‐fat diets (HFD) identified as a primary modifiable risk factor (Xiang et al. 2024; Younossi et al. 2025). Consequently, dietary lipid excess is now recognized as a pivotal target for primary intervention strategies (Beygi et al. 2024).

Chronic HFD intake disrupts hepatic lipid homeostasis through coordinated dysregulation of lipogenesis, fatty acid oxidation (FAO), and lipid export pathways. Mechanistically, HFD activates lipogenic transcription factors to enhance *de novo* lipogenesis, suppresses mitochondrial β‐oxidation, and dysregulates circadian lipid metabolism (Steinberg et al. 2025). Lipid overload subsequently progresses to hepatocyte injury via lipotoxicity, wherein saturated free fatty acids (FFAs) induce mitochondrial dysfunction and endoplasmic reticulum stress, thereby activating NF‐κB pathways that recruit pro‐inflammatory macrophages and amplify hepatic inflammation (Iturbe‐Rey et al. 2025; Venkatesan et al. 2023). Notably, accumulating evidence confirms gut dysbiosis as a critical mechanistic link between HFD and MASLD pathogenesis (Mignini et al. 2024). Specifically, HFD enriches pathogenic taxa ( *Bacteroides fragilis* and *Desulfovibrionaceae*), elevating endotoxin lipopolysaccharide (LPS) production. The resultant LPS translocates across compromised intestinal tight junctions, thereby activating hepatic TLR4/MyD88 signaling and TNF‐α‐driven inflammation (Huang et al. 2024). Concurrently, HFD depletes beneficial genera including *Akkermansia muciniphila* , *Lactobacillus*, and *Bifidobacterium*; this reduction diminishes short‐chain fatty acid (SCFA) production, impairing both intestinal barrier integrity and PPARα‐dependent FAO in hepatocytes (Liu et al. 2025). Consequently, gut microbiota‐targeted interventions such as fecal microbiota transplantation, probiotic supplementation, and time‐restricted feeding regimens demonstrate efficacy in ameliorating MASLD by restoring microbial balance, enhancing barrier function, and correcting lipid dysmetabolism; these approaches collectively attenuate hepatic lipotoxicity, inflammation, and fibrosis, offering promising translational strategies for metabolic liver diseases (Nie et al. 2024; Zhang and Brandman 2025).

*Panax notoginseng* (Burk.) F.H. Chen (Araliaceae family) is a botanically sourced material recognized for its long history of use and excellent medicinal and food dual‐use characteristics (Wang et al. 2016). The root‐derived *Panax notoginseng* saponins (PNS), which constitute the primary bioactive components, have been widely employed as nutritional supplements for many years (Xu et al. 2019). PNS are known for their multi‐faceted regulatory properties, such as promoting hemostasis while simultaneously inhibiting thrombosis and improving microcirculation (Zhang, Wang, et al. 2025). Such functional versatility aligns with their historical use in managing bleeding disorders and conditions related to blood stasis. Modern research further supports the broad bioactivities of PNS, including roles in cardiovascular protection, neuroregulation, inflammation modulation, and antitumor potential (Li, Shi, and Wu 2025), with reported benefits in experimental models of hepatic fibrosis and liver injury (Hui et al. 2016; Tian et al. 2024). Despite these documented effects, the protective role and underlying mechanisms of PNS in the context of hyperlipidemia‐induced liver injury remain insufficiently explored. Therefore, this study aims to investigate the hepatoprotective effects of PNS using a high‐fat diet (HFD)‐induced mouse model and to delineate the involved mechanisms through an integrated approach combining in vivo analysis with in vitro pharmacological inhibition in LPS‐stimulated hepatocytes.

## 2 Material and Methods

### 2.1 Chemicals and Reagents

PNS (purity ≥ 95%) was purchased from Solarbio Life Sciences (Beijing, China). Antibodies against TLR4, MyD88, Erk1/2, phospho‐Erk1/2 (Thr^202^/Tyr^204^), p38 MAPK, phospho‐p38 MAPK (Thr^180^/Tyr^182^), JNK, and phospho‐JNK (Thr^183^/Tyr^185^) were purchased from Affinity Biosciences (OH, USA). Antibodies against NF‐κB p65, phospho‐NFκB p65 (Ser^536^), phospho‐IκBα (Tyr^42^), IκBα, GAPDH and β‐actin were purchased from Bioworld Technology (MN, USA). Secondary antibodies including horseradish peroxidase (HRP)‐conjugated goat anti‐rabbit IgG and anti‐mouse IgG were purchased from Servicebio (Wuhan, China). TAK‐242 was obtained from Selleck Chemical (Houston, Texas, USA). LPS with a specific activity of ≥ 500,000 EU/mg was purchased from Solarbio (Beijing, China).

### 2.2 Animals and Diets

Male C57BL/6J mice (6 weeks old, Kunming Chushang Technology Co. Ltd., China) were housed under specific‐pathogen‐free (SPF) conditions at the Dali University Animal Facility with controlled temperature (22°C–24°C) and a 12‐h light–dark cycle. After 2 weeks of acclimatization with *ad libitum* access to food and water, mice were randomly assigned to four dietary groups. These groups were fed for 12 weeks with either a low‐fat diet (LFD) containing 10% fat (LFD group), a LFD supplemented with PNS at a concentration of 200 mg/kg diet (LFD + PNS group), a HFD containing 45% fat (HFD group), or a HFD supplemented with PNS at the same concentration (HFD + PNS group). PNS powder (Medicience Ltd., China) was homogeneously blended into respective diets, with detailed compositions provided in Table S1. Body weight and food intake were recorded weekly. The PNS dosage used in this study was determined based on our previous investigation (Ma et al. 2026) and is consistent with an established protocol reported in the literature (Zhang et al. 2021). All procedures were approved by the Dali University Animal Ethics Committee (SYXK [Dian] 2018‐0002).

### 2.3 Blood and Liver Tissue Collection

All experimental procedures were conducted in accordance with the ARRIVE guidelines and the American Veterinary Medical Association (AVMA) Guidelines for the Euthanasia of Animals (2020). Mice were deeply anesthetized using isoflurane, delivered at a concentration of 3% in an anesthesia chamber for induction, followed by maintenance at 1.5%–2% via a nose cone with oxygen as the carrier gas. Subsequently, a lethal overdose of pentobarbital sodium (150 mg/kg body weight) was administered via intraperitoneal injection. Death was confirmed by the absence of pedal and other reflexes prior to tissue collection. No signs of peritonitis or notable abdominal distress were observed during the procedure.

Blood was collected via cardiac puncture into tubes containing a 1/10 volume of 3.8% acid citrate dextrose (38 mM citric acid, 75 mM trisodium citrate, 100 mM dextrose). The anticoagulated blood was centrifuged at 300 × *g* for 2 min at 37°C to obtain platelet‐rich plasma (PRP). The PRP was further centrifuged at 500 × *g* for 3 min, and the resulting supernatant (platelet‐poor plasma, PPP) was aliquoted and stored at −80°C for subsequent analysis (Ma et al. 2026). Following blood collection, the thoracic cavity was opened and the vasculature was perfused with saline via the left ventricle to clear residual blood. Liver tissues were then excised, weighed, and stored appropriately for subsequent experiments.

### 2.4 Hematoxylin–Eosin (HE) Staining

HE staining was performed as previously described (Cao et al. 2024; Liu, Wang, et al. 2024; Ma et al. 2024). Briefly, freshly harvested liver was fixed in 4% paraformaldehyde (PFA, pH 7.2) and embedded in paraffin. Liver sections (5 μm in thickness) were stained with HE for morphological evaluation. Sections were digitalized using a Leica microscope (Leica Microsystems, Heidelberg, Germany).

### 2.5 Oil Red O Staining

Liver tissues were harvested, embedded in optimal cutting temperature (OCT) compound, and immediately flash‐frozen in liquid nitrogen. Serial cryosections (5 μm thickness) were prepared using a cryostat (Leica CM1950), fixed in 10% neutral buffered formalin for 10 min at room temperature, and rinsed with distilled deionized water. Sections were incubated with pre‐filtered (0.45 μm) Oil Red O working solution (0.5% w/v in isopropanol) for 30 min at 37°C, followed by differentiation in 60% isopropanol for 15 s to remove non‐specific staining. Nuclei were counterstained with Mayer's hematoxylin for 1 min, and sections were mounted with aqueous mounting medium. Lipid droplets were visualized as red intracellular deposits under bright‐field microscopy (Leica Microsystems, Heidelberg, Germany). Quantitative analysis was performed using ImageJ software (version 1.37v; NIH, USA) by calculating the percentage of Oil Red O‐positive area relative to total hepatic parenchymal area across five randomly selected fields per section in three independent experiments.

### 2.6 Measurement of Serum Lipid Profiles

The levels of total cholesterol (TC), triglycerides (TG), high‐density lipoprotein cholesterol (HDL‐C), and low‐density lipoprotein cholesterol (LDL‐C) in the prepared platelet‐poor plasma samples (see Section 2.3) were quantified using commercial assay kits (Nanjing Jiancheng Bioengineering Institute, China) in accordance with the manufacturer's protocols.

### 2.7 In Vitro Cell Culture and Treatment

The human hepatocellular carcinoma cell line HepG2 was obtained from the American Type Culture Collection (ATCC). Cells were cultured in Dulbecco's Modified Eagle Medium (DMEM) supplemented with 10% fetal bovine serum (FBS) and 1% penicillin–streptomycin at 37°C in a humidified atmosphere containing 5% CO_2_.

For drug treatments, cells were seeded in appropriate culture plates and allowed to adhere overnight. To investigate the protective effect of PNS, cells were pretreated with various concentrations of PNS (1, 10, and 100 μg/mL) for 24 h. Following pretreatment, the culture medium was removed and replaced with fresh medium containing lipopolysaccharide (LPS, 1 μg/mL) for an additional 24 h to induce inflammatory injury. To pharmacologically validate the involvement of the TLR4 pathway, a parallel experiment was performed using the specific TLR4 inhibitor TAK‐242 (5 μM). In the combination treatment group, cells were co‐treated with PNS (100 μg/mL) and TAK‐242 (5 μM) during the LPS challenge phase. Following the treatments, cells and culture supernatants were collected for subsequent analyses of cell viability, oxidative stress markers, inflammatory cytokines, and protein expression.

### 2.8 Cell Viability Assay

Cell viability was assessed using a Cell Counting Kit‐8 (CCK‐8; Solarbio, Beijing, China) according to the manufacturer's instructions to evaluate the potential cytotoxic effects of PNS and LPS. Briefly, HepG2 cells were seeded in 96‐well plates. After adherence, cells were pretreated with various concentrations of PNS (1, 10, and 100 μg/mL) or vehicle control for 24 h. The medium was then replaced with fresh medium containing LPS (1 μg/mL) for an additional 24‐h challenge. Following the total 48‐h treatment period, 10 μL of CCK‐8 reagent was added to each well and incubated for 2 h at 37°C. The optical density of each well was measured at a wavelength of 450 nm using a microplate reader. Cell viability for each group was calculated based on its OD value and expressed as a percentage.

### 2.9 Analysis of Hepatic Transaminases and Inflammatory Cytokines

Aspartate aminotransferase (AST) and alanine aminotransferase (ALT) levels were quantified using commercial kits (Beijing Boxbio Science and Technology Co. Ltd., China). Briefly, 5 μL serum supernatant was aliquoted into microplate wells followed by addition of working reagent. After sequential incubation steps, 250 μL chromogenic substrate was dispensed to initiate enzymatic reactions. Plates were incubated at room temperature for 10 min with gentle agitation, and absorbance was measured at 505 nm. Concurrently, hepatic pro‐inflammatory cytokines including interleukin‐1β (IL‐1β; Solarbio Life Sciences, China) and tumor necrosis factor‐α (TNF‐α; Cloud‐Clone Corp., Wuhan, China) were assayed using enzyme‐linked immunosorbent assay (ELISA) kits according to manufacturers' protocols.

The levels of AST, ALT, TNF‐α, and interleukin‐6 (IL‐6) in the culture supernatants of HepG2 cells were also determined. AST and ALT activities were measured using the same commercial kits as for serum samples, with appropriate dilution of the supernatant. The concentrations of TNF‐α and IL‐6 in the supernatant were quantified using specific human ELISA kits (Cloud‐Clone Corp., Wuhan, China) strictly following the manufacturers' instructions.

### 2.10 Hepatic and Cellular Oxidative Stress Evaluation

Total antioxidant capacity (T‐AOC), superoxide dismutase (SOD) activity, reduced glutathione (GSH), malondialdehyde (MDA) levels, and the reduced to oxidized glutathione (GSH/GSSG) ratio in liver tissue were measured using respective assay kits (Beijing Boxbio Science and Technology Co. Ltd., China). Liver samples (100 mg) were homogenized in ice‐cold PBS and centrifuged at 10,000 × *g* for 10 min at 4°C. Supernatant protein concentrations were determined prior to analysis. Absorbance was measured at 593 nm (T‐AOC), 560 nm (SOD), 412 nm (GSH), and 532 nm (MDA). Results were expressed as μmol/g tissue (T‐AOC), U/g tissue (SOD), μg/g tissue (GSH and GSSG), and nmol/L (MDA). The GSH/GSSG ratio was calculated based on the measured levels of reduced GSH and oxidized glutathione (GSSG).

The oxidative stress status in HepG2 cells was assessed by measuring intracellular levels of MDA, SOD, GSH, and the GSH/GSSG ratio. After treatments, cells were washed, lysed, and centrifuged to obtain the supernatant. The protein concentration of the lysate was determined. MDA content (nmol/mg protein), SOD activity (U/mg protein), and GSH content (U/mg protein) were measured using the respective commercial assay kits (Beijing Boxbio Science and Technology Co. Ltd., China) according to the manufacturers' protocols, with absorbance readings at the specified wavelengths.

### 2.11 Quantification of LPS in Serum and Liver Tissue

LPS concentrations were measured with a chromogenic TAL assay kit (Solarbio, Beijing, China). Serum was obtained from whole blood by centrifugation. Liver tissues were homogenized in pyrogen‐free PBS, and the supernatants were collected after centrifugation (see Section 2.3). Following sample heat‐inactivation (70°C, 10 min), the assay was conducted per the kit protocol. Absorbance was read at 545 nm, and LPS levels were calculated against a standard curve. Hepatic LPS levels were normalized to total protein content determined by a BCA assay. Results are presented as EU/mL (serum) and EU/mg protein (liver).

### 2.12 Western Blotting Analysis

Immunoblotting was performed according to our previously described methods (Bi et al. 2025; Li, Wu, et al. 2025; Zhou et al. 2023). Briefly, liver tissues and HepG2 cells were homogenized in RIPA lysis buffer with protease/phosphatase inhibitors (Beyotime Biotechnology, China) using a tissue homogenizer (Servicebio, China). Lysates were centrifuged (12,000 × *g*, 15 min, 4°C), and supernatants subjected to protein quantification via BCA assay (KeyGEN Biotechnology, China). Aliquots (30 μg total protein) were denatured, separated by SDS‐PAGE, and electrotransferred to PVDF membranes (Millipore, USA). After blocking with 5% BSA (1.5 h), membranes were incubated overnight at 4°C with primary antibodies. Following three TBST washes, membranes were incubated (1.5 h) with HRP‐conjugated secondary antibodies. After additional washes, protein bands were visualized using ECL detection. Band intensities were quantified with ImageJ (version 1.37v; NIH, USA) and normalized to β‐actin or GAPDH.

### 2.13 Sample Size, Exclusion Criteria, and Statistical Analysis

Sample size and justification: The sample sizes for key endpoints (e.g., body weight, liver weight, serum lipids, *n* = 10) were determined based on previous studies in our lab (Ma et al. 2026), which typically show large effect sizes. For some exploratory or resource‐intensive assays (e.g., specific hepatic cytokines, oxidative stress markers in vitro), initial sample sizes were smaller but were found to be sufficient to detect statistically significant differences between the HFD and HFD + PNS groups. No formal a priori power calculation was performed for all endpoints, as this study was designed as a comprehensive mechanistic investigation.

Exclusion criteria: No animals or data points were excluded from the analysis. All mice that completed the 12‐week intervention were included in all analyses for which tissue/samples were available.

Statistical analysis: Data from ≥ 3 independent biological replicates were expressed as means with standard deviation (mean ± SD). Statistical significance was assessed by one‐way analysis of variance (ANOVA) followed by Dunnett's or Tukey's multiple comparisons test using GraphPad Prism software (version 9.4.1; GraphPad Inc., San Diego, CA, USA). Spearman's rank correlation analysis assessed relationships between differential lipids and biological parameters. A threshold of *p* < 0.05 defined statistical significance.

### 2.14 Untargeted Lipidomics Analysis by UPLC‐TOF/MS

#### 2.14.1 Sample Preparation and Mass Spectrometric Conditions

Liver tissues (50 mg) were homogenized in 200 μL ice‐cold 50% methanol using a Precellys 24 homogenizer (JXFSTPRP‐CLN, China) with 3.0 mm steel beads (6000 rpm, 30 s × 2 cycles, 4°C), followed by addition of 600 μL methyl tert‐butyl ether (MTBE) and vortex‐mixing (30 min, 4°C). After centrifugation (3000 × *g*, 15 min, 4°C), 200 μL of the organic phase was lyophilized (Labconco CentriVap, USA), reconstituted in 200 μL dichloromethane: methanol (1:1, v/v) containing 0.01% butylated hydroxytoluene (BHT), and recentrifuged (3000 × *g*, 15 min, 4°C); supernatants were transferred to vials, with quality control (QC) samples prepared from pooled aliquots. Chromatographic separation used an ACQUITY UPLC CSH C18 column (100 × 2.1 mm, 1.7 μm; 40°C) at 0.3 mL/min with mobile phase A (10 mM ammonium formate/0.1% formic acid in acetonitrile: water 60:40) and B (10 mM ammonium formate/0.1% formic acid in isopropanol: acetonitrile 90:10) under gradient elution: 0–0.4 min (30% B), 0.4–1.0 min (30%→45% B), 1.0–3.5 min (45%→60% B), 3.5–5.0 min (60%→75% B), 5.0–7.0 min (75%→90% B), 7.0–8.5 min (90%→100% B), 8.5–8.6 min (100% B), 8.6–8.61 min (100%→30% B), 8.61–10.0 min (30% B).

Mass spectrometry was performed on a high‐resolution tandem mass spectrometer TripleTOFTM 6600 system (SCIEX, NL) equipped with DuoSpray ion source. Data acquisition in both positive (+5000 V) and negative (−4500 V) electrospray ionization (ESI) modes used the following parameters: source temperature 500°C, curtain gas 30 psi, nebulizer/heater gases 60 psi, and declustering potential 80 V. Mass spectrometric data were acquired using a combined full‐scan and information‐dependent acquisition (IDA) approach, where each acquisition cycle initiated with full‐scan MS (*m/z* 50–2000, 170 ms accumulation), followed by real‐time selection of the top 12 ions exceeding 100 cps intensity for IDA‐triggered MS/MS scans (*m/z* 25–1200, 30 ms accumulation) incorporating 4 s dynamic exclusion.

#### 2.14.2 Lipidomics Quality Control

Quality control (QC) pools (*n* = 6) were injected at every sixth sample interval to monitor system stability. QC procedures comprised total ion chromatogram (TIC) stability assessment, high‐quality feature selection using K‐nearest neighbors (KNN) imputation with probabilistic quotient normalization (PQN) and coefficient of variation (CV) filtering (CV < 30% threshold), multivariate pattern verification through principal component analysis (PCA) and hierarchical clustering, and correlation evaluation. All batches met validation criteria, including inter‐QC Pearson's correlation *r* > 0.95 and PCA dispersion accounting for < 2% total variance.

#### 2.14.3 Lipidomic Data Processing and Analysis

LC–MS raw data converted to mzML format were processed using XCMS in R (v4.1.1) for peak picking, retention time alignment, isotope/adduct annotation, and feature grouping. A three‐dimensional matrix of peak indices, sample names, and ion intensities was constructed from retention time and *m/z*‐matched ions. Metabolite annotation via KEGG and HMDB databases employed precise *m/z* matching. After preprocessing in R, group comparisons utilized one‐way ANOVA with Tukey's post hoc test (*α* = 0.05), applying Benjamini‐Hochberg false discovery rate (FDR) correction. Discriminatory features were identified by partial least squares‐discriminant analysis (PLS‐DA), with biological relevance assigned to features exhibiting variable importance in projection (VIP) scores > 1.0.

## 3 Results

### 3.1 PNS Alleviated HFD‐Induced Liver Injury in Mice

**FIGURE 1:**
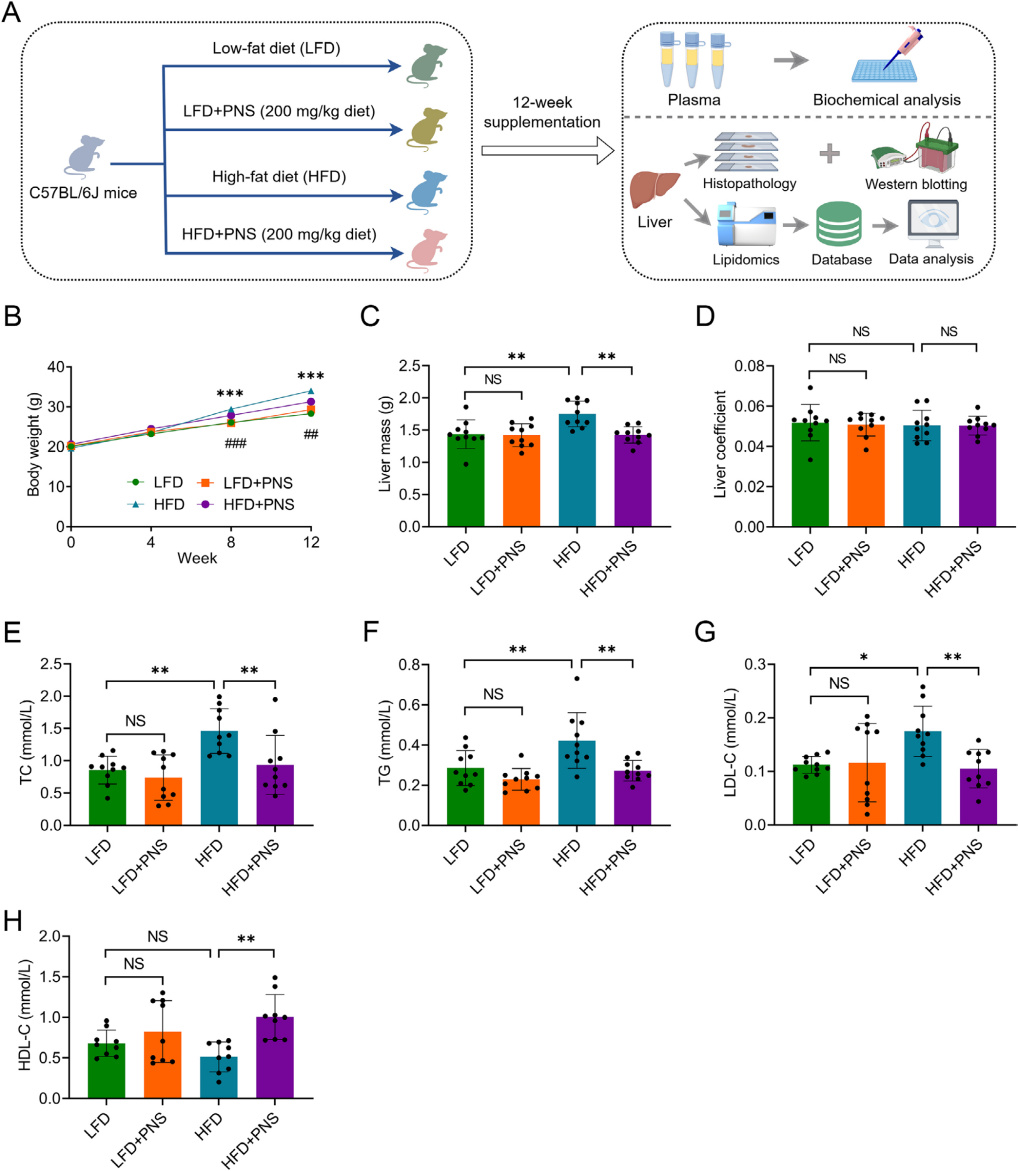
PNS alleviate dyslipidemia in HFD‐fed mice. (A) Schematic diagram of the experimental design. (B) Body weight changes in mice from the LFD, LFD + PNS, HFD, and HFD + PNS groups throughout the study. ***p < 0.001: LFD vs. HFD group; ^##^ *p* < 0.01 and ^###^ *p* < 0.001: HFD vs. HFD + PNS group (*n* = 10). (C, D) Absolute liver weight (C) and liver‐to‐body weight ratio (D) at the endpoint (*n* = 10). (E–H) Serum lipid parameters, including TC (E), TG (F), LDL‐C (G) and HDL‐C (H) (*n* = 10 except *n* = 9 in H). Data were assessed by one‐way ANOVA followed by Tukey's multiple comparisons test. *p < 0.05 and **p < 0.01; NS, not significant.

Multimodal analyses demonstrated that PNS supplementation effectively attenuated HFD‐induced hepatic injury. Throughout the 12‐week intervention (Figure 1A), HFD‐fed mice developed a significant increase in body weight starting from week 8 compared to LFD controls, an effect that was mitigated by PNS co‐administration (Figure 1B). Consistent with the systemic phenotype, the absolute liver weight was significantly higher in the HFD group than in the LFD controls but was reduced by PNS treatment (Figure 1C); however, the liver‐to‐body weight ratio remained comparable across all groups (Figure 1D). Moreover, PNS supplementation favorably modulated the dysregulated serum lipid profile in HFD‐fed mice, as evidenced by decreased levels of TC, TG, and LDL‐C, alongside an increase in HDL‐C (Figure 1E–H).

**FIGURE 2:**
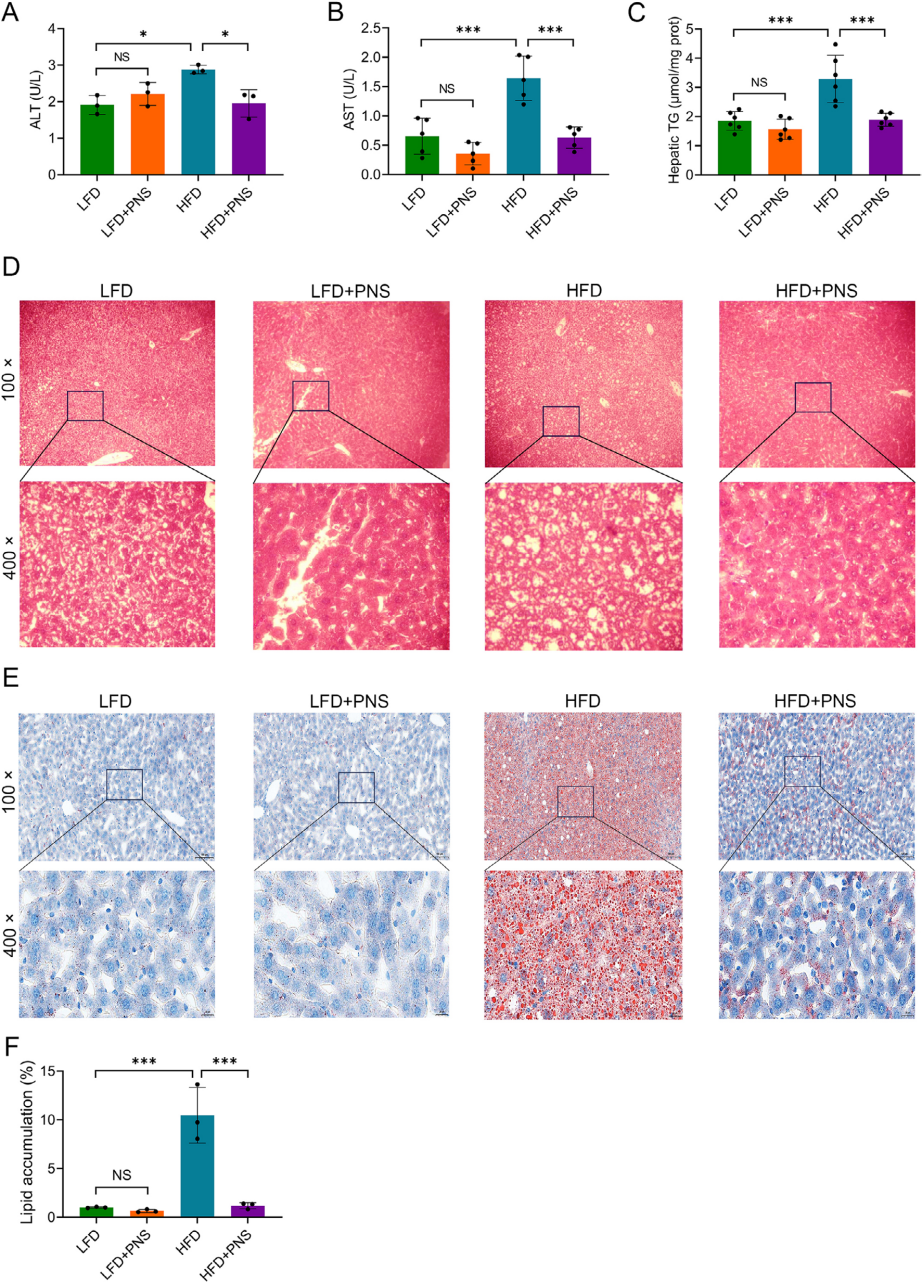
PNS ameliorate HFD‐induced liver injury in mice. (A, B) Plasma levels of ALT (*n* = 3) (A) and AST (B) (*n* = 5). (C) Hepatic TG content (*n* = 6). (D) Representative images of H&E‐stained liver sections. Upper panel: Overview (scale bar, 50 μm, 100× magnification). Lower panel: Detailed views (scale bar, 10 μm, 400× magnification). (E) Representative images of Oil Red O‐stained liver sections. Upper panel: Overview (scale bar, 100 μm, 100× magnification). Lower panel: Detailed views (scale bar, 20 μm, 400× magnification). (F) Quantification of the Oil Red O‐positive area relative to the total parenchymal area (*n* = 3). Data were assessed by one‐way ANOVA followed by Tukey's multiple comparisons test. *p < 0.05 and ***p < 0.001; NS, not significant.

At the biochemical level, PNS treatment significantly reversed the HFD‐induced elevation in the serum activities of the hepatic injury markers ALT and AST (Figure 2A,B). Concordantly, the increase in hepatic TG content induced by HFD was also markedly attenuated by PNS (Figure 2C). Histopathological examination of liver sections revealed well‐preserved hepatic architecture in both the LFD and LFD + PNS groups. In contrast, livers from HFD‐fed mice displayed substantial pathological alterations, including irregular lobular contours, hepatocyte ballooning, and inflammatory cell infiltration (Figure 2D). These structural abnormalities were substantially ameliorated by PNS co‐administration. Furthermore, Oil Red O staining confirmed pronounced hepatic lipid accumulation, characterized by extensive lipid vacuolation, in HFD‐fed mice, which was markedly reduced upon PNS treatment (Figure 2E,F). Collectively, these findings indicate that PNS supplementation effectively alleviates HFD‐induced liver injury in mice.

### 3.2 PNS Attenuated HFD‐Induced Hepatic Oxidative Stress and Inflammation

**FIGURE 3:**
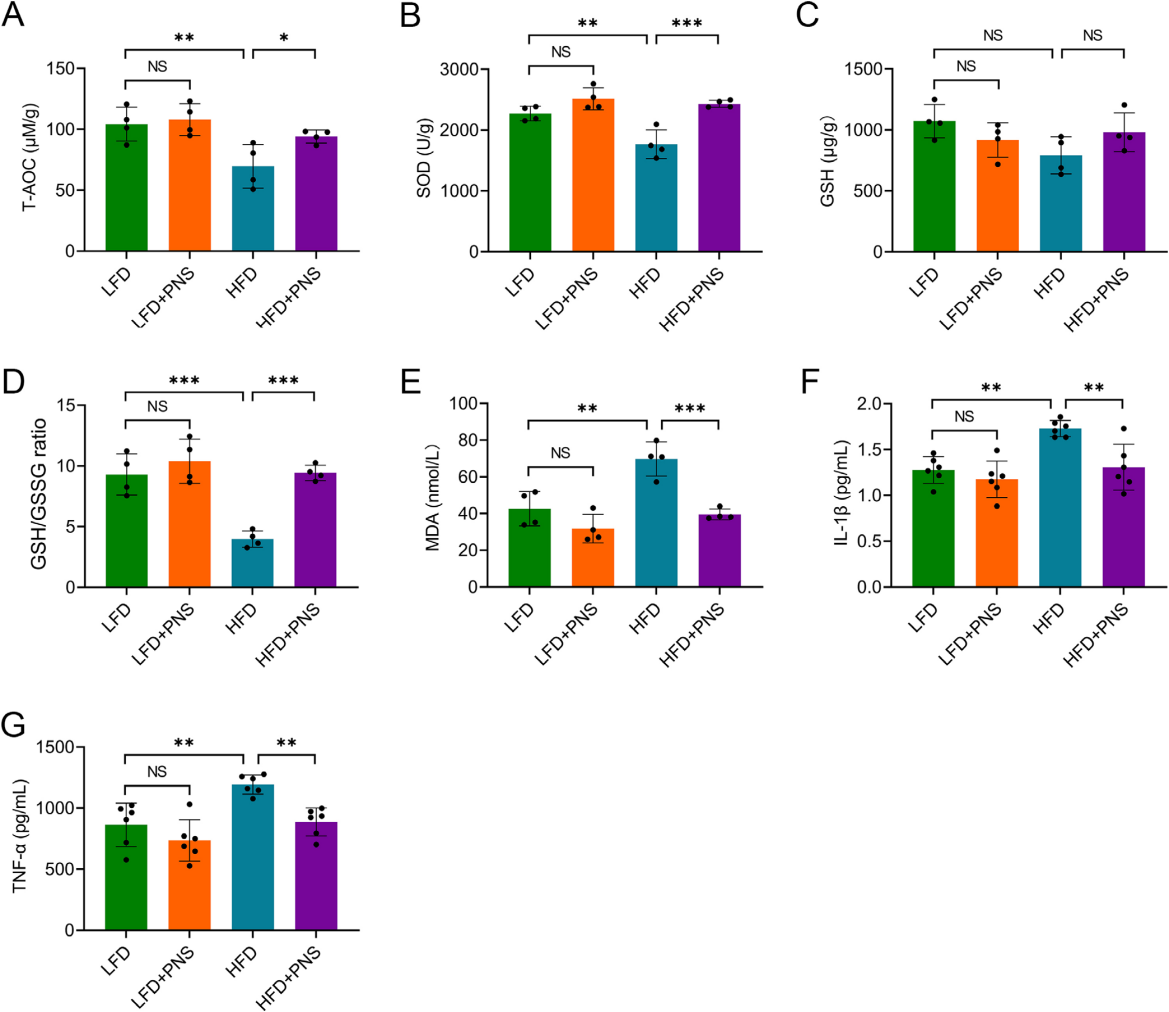
PNS attenuate hepatic oxidative stress and inflammation in HFD‐fed mice. (A–E) Hepatic oxidative stress parameters, including T‐AOC (A), SOD activity (B), GSH level (C), GSH/GSSG ratio (D), and MDA content (E) (*n* = 4). (F, G) Hepatic concentrations of the pro‐inflammatory cytokines IL‐1β (F) and TNF‐α (G) (*n* = 6). Data were assessed by one‐way ANOVA followed by Tukey's multiple comparisons test. *p < 0.05, **p < 0.01 and ***p < 0.001; NS, not significant.

To investigate the impact of PNS on hepatic oxidative stress and inflammation, we analyzed key biomarkers in experimental groups. Compared to LFD controls, HFD‐fed mice exhibited significantly reduced hepatic T‐AOC (Figure 3A) and SOD activity (Figure 3B). Notably, 12‐week PNS supplementation effectively restored these parameters in HFD mice. In contrast, neither HFD nor PNS intervention significantly altered hepatic GSH levels (Figure 3C). Critically, PNS treatment attenuated HFD‐induced lipid peroxidation, as evidenced by reduced MDA concentrations (Figure 3D). Concurrently, HFD feeding markedly upregulated pro‐inflammatory cytokines IL‐1β and TNF‐α, while PNS co‐administration significantly suppressed their expression (Figure 3E,F). In contrast, PNS supplementation did not alter hepatic oxidative stress or inflammation markers in LFD mice. These results collectively demonstrate that PNS specifically mitigate HFD‐induced hepatic oxidative stress and inflammation.

### 3.3 Non‐Targeted Lipidomic Analysis of the Livers

**FIGURE 4:**
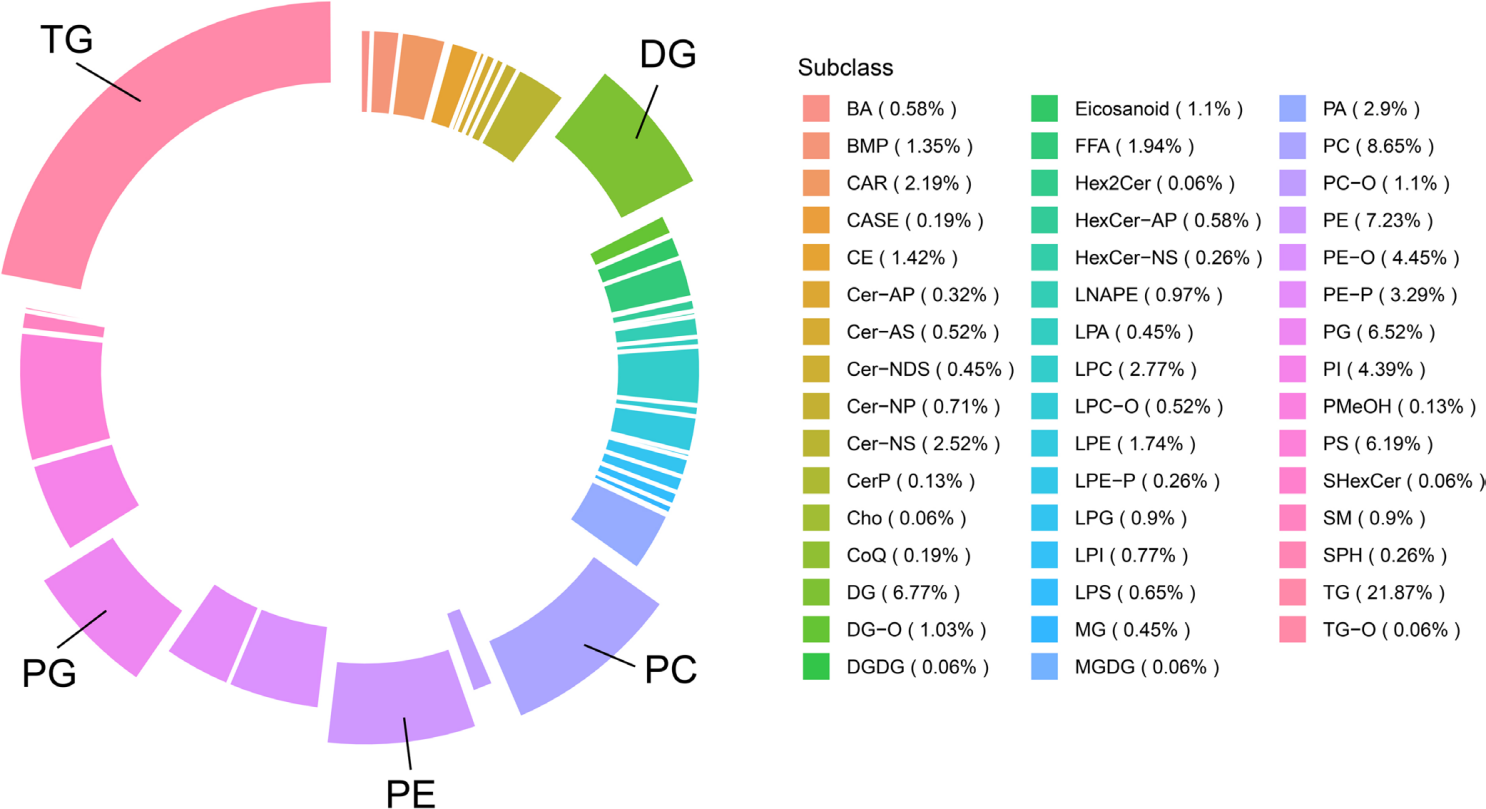
Composition and distribution of hepatic lipid subclasses. The relative abundance of all identified lipid subclasses across the experimental groups was displayed.

**FIGURE 5:**
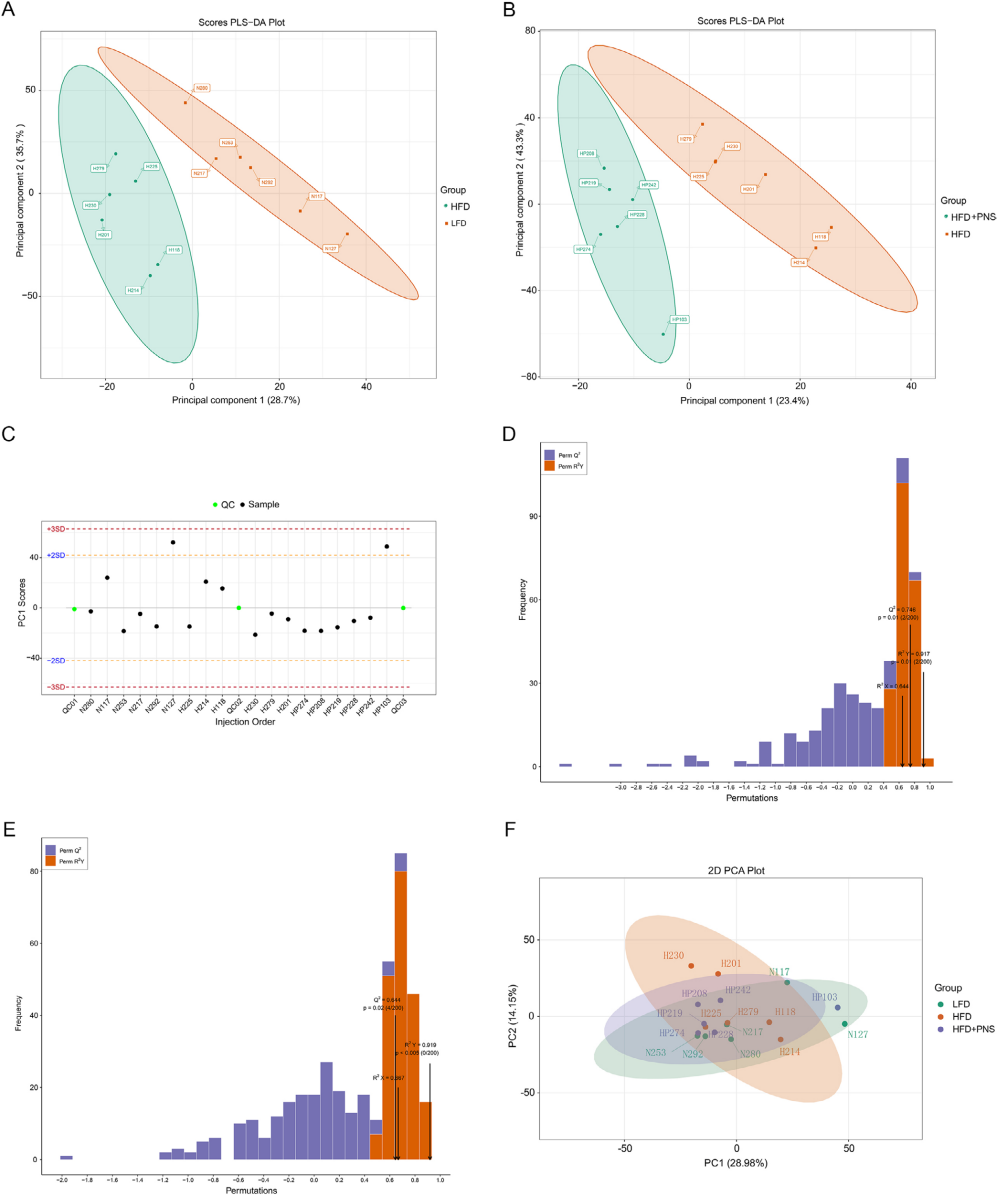
Untargeted lipidomics profiling of liver tissues by UPLC‐TOF/MS. (A, B) The PLS‐DA score plots depicting the metabolic profiles of the LFD, HFD, and HFD + PNS groups. (C) Distribution of PC1 scores, confirming all samples fell within ±3 standard deviations. (D–E) Validation of the orthogonal PLS‐DA model for the HFD vs. LFD comparison (D) and the HFD vs. HFD + PNS comparison (E). (F) Principal component analysis of the lipidomics data.

Non‐targeted hepatic lipidomic profiling via UPLC‐TOF/MS quantified 1601 lipid compounds after rigorous quality control (relative standard deviation < 30%), with 829 features detected in ESI^+^ and 772 in ESI^−^. These lipids spanned 47 distinct subclasses, dominated by TG (21.87%), phosphatidylcholines (PC, 8.65%), phosphatidylethanolamines (PE, 7.23%), diacylglycerols (DG, 6.67%), and phosphatidylglycerols (PG, 6.52%); complete subclass distributions were documented in Figure 4. Partial least squares‐discriminant analysis (PLS‐DA) revealed pronounced metabolic separation between dietary groups. LFD versus HFD cohorts and HFD versus HFD + PNS cohorts exhibited discrete clustering in score plots (Figure 5A,B), with minimal intra‐group dispersion indicating biological consistency. Analytical robustness was confirmed through multiple validation strategies. Principal component stability metrics showed that the PC1 scores fell within the range of plus or minus three standard deviations (3SD) (Figure 5C), indicating instrument reproducibility. Moreover, the orthogonal PLS‐DA models demonstrated high explanatory power (HFD vs. LFD: *R* ^2^Y = 0.917, *Q* ^2^ = 0.746; HFD vs. HFD + PNS: *R* ^2^Y = 0.919, *Q* ^2^ = 0.644; Figure 5D,E). Principal component analysis (PCA) further captured 43.13% cumulative variance through PC1 and PC2 components (Figure 5F). This convergent evidence establishes model reliability without overfitting.

**FIGURE 6:**
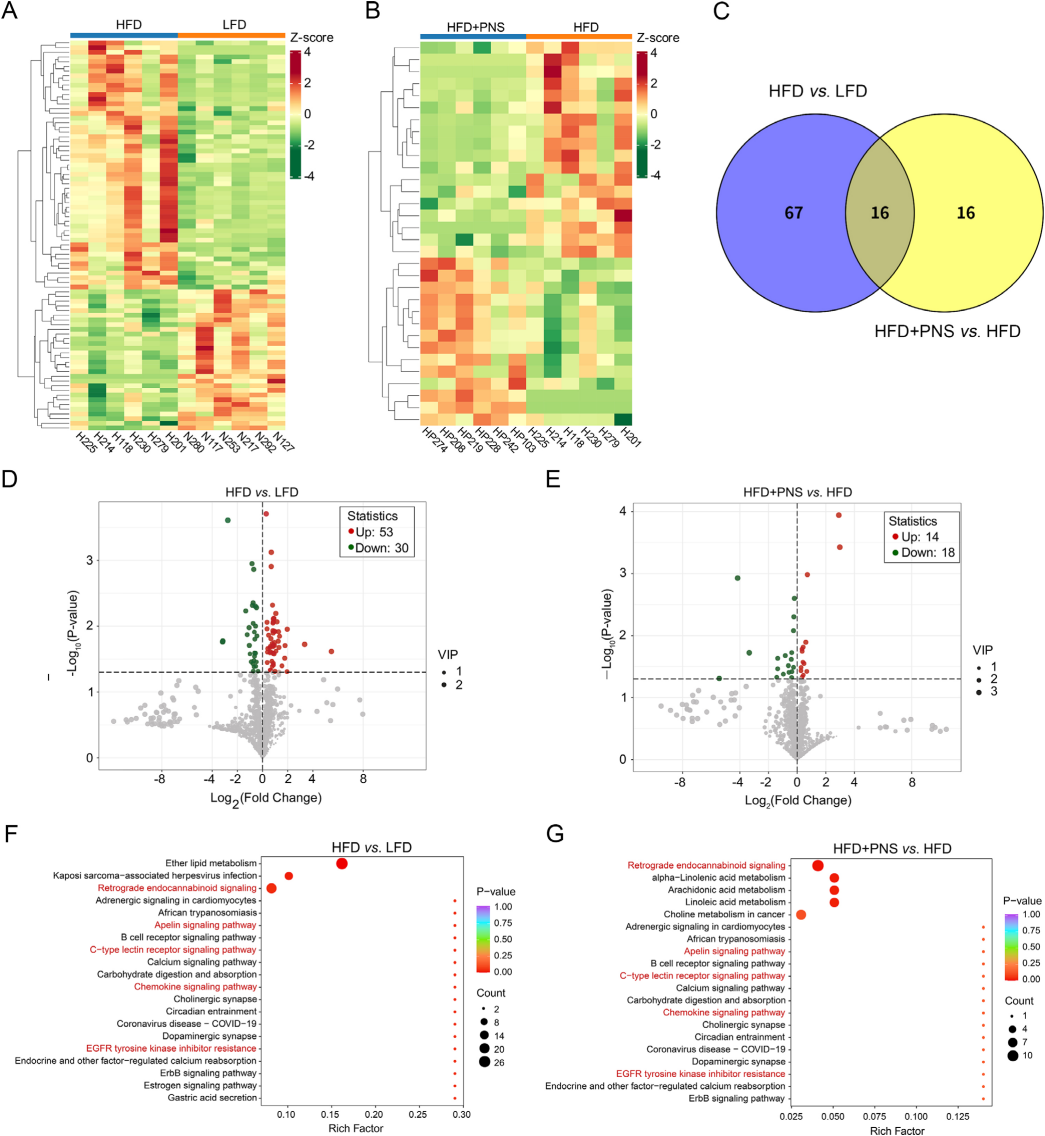
Identification of differential lipid species. (A, B) Heatmaps visualizing significantly altered lipids between the LFD and HFD groups (A), and between the HFD and HFD + PNS groups (B). (C) Venn diagram illustrating the overlap of differential lipids among the three groups. (D–E) Volcano plots showing lipid species with significant changes (fold change and statistical significance) in the LFD vs. HFD comparison (D) and the HFD vs. HFD + PNS comparison (E). (F, G) KEGG pathway enrichment analysis based on differential lipids from the LFD vs. HFD comparison (F) and the HFD vs. HFD + PNS comparison (G). Significantly enriched pathways were identified based on *p*‐values and Rich Factor.

Hierarchical clustering analysis (VIP > 1.0, *p* < 0.05) identified 177 hepatic lipids with differential abundance, as annotated using the KEGG and HMDB databases. Distinct lipid profiles were observed in the heatmap visualization when comparing the LFD group to the HFD group, as well as the HFD group to the HFD + PNS groups (Figure 6A,B). Venn diagram analysis revealed that, compared to the LFD group, 83 lipids were significantly altered in the HFD group; supplementation with PNS significantly modulated 32 lipids in HFD‐fed mice, with 16 lipids commonly regulated across comparisons (Figure 6C). Volcano plots further quantified these changes: relative to the LFD group, the HFD group showed 53 upregulated and 30 downregulated lipids (Figure 6D). In contrast, the HFD + PNS group exhibited 14 upregulated and 18 downregulated lipid species compared to the HFD group (Figure 6E). KEGG pathway enrichment analysis indicated that the HFD induced dysregulation in lipid metabolism‐associated signaling pathways, including retrograde endocannabinoid, apelin, C‐type lectin receptor, and chemokine, and EGFR tyrosine kinase inhibitor resistance pathways (Figure 6F). These pathways were significantly normalized following PNS intervention (Figure 6G), indicating a systemic restoration of hepatic lipid homeostasis.

**FIGURE 7:**
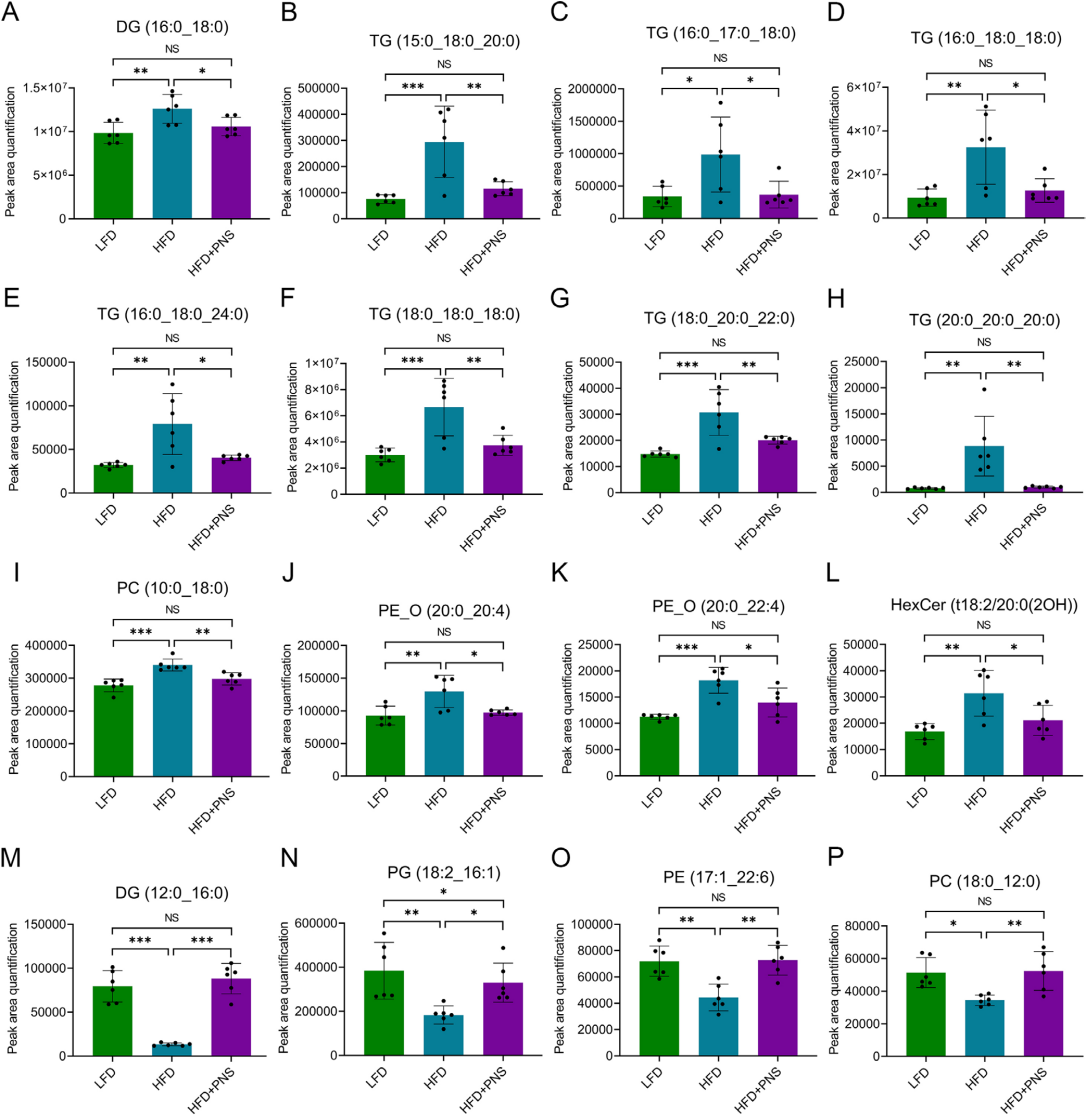
Quantitative analysis of differential lipids among experimental groups. (A–P) Relative abundances of significantly altered hepatic lipids, as quantified by peak area from UPLC‐TOF/MS analysis, are shown for the LFD, HFD, and HFD + PNS groups (*n* = 6). Data were assessed by one‐way ANOVA followed by Tukey's multiple comparisons test. *p < 0.05, **p < 0.01 and ***p < 0.001; NS, not significant.

Among the 16 commonly regulated lipids, compared to the LFD group, the HFD group exhibited significant upregulation of DG (16:0_18:0), TG (15:0_18:0_20:0), TG (16:0_17:0_18:0), TG (16:0_18:0_18:0), TG (16:0_18:0_24:0), TG (18:0_18:0_18:0), TG (18:0_20:0_22:0), TG (20:0_20:0_20:0), PC (10:0_18:0), 1‐alkyl, 2‐acylglycerophosphatidylethanolamines (PE_O) (20:0_20:4), PE_O (20:0_22:4), and hexosylceramide (HexCer) (d18:2/20:0 (2OH)) (Figure 7A–L), along with downregulation of DG (12:0_16:0), PG (18:2_16:1), PE (17:1_22:6), and PC (18:0_12:0) (Figure 7M–P). Notably, these HFD‐induced alterations were markedly restored by PNS supplementation in HFD‐fed mice (Figure 7).

### 3.4 PNS Suppressed Hepatic TLR4/MyD88‐Mediated NF‐κB and MAPK Signaling in HFD‐Fed Mice

**FIGURE 8:**
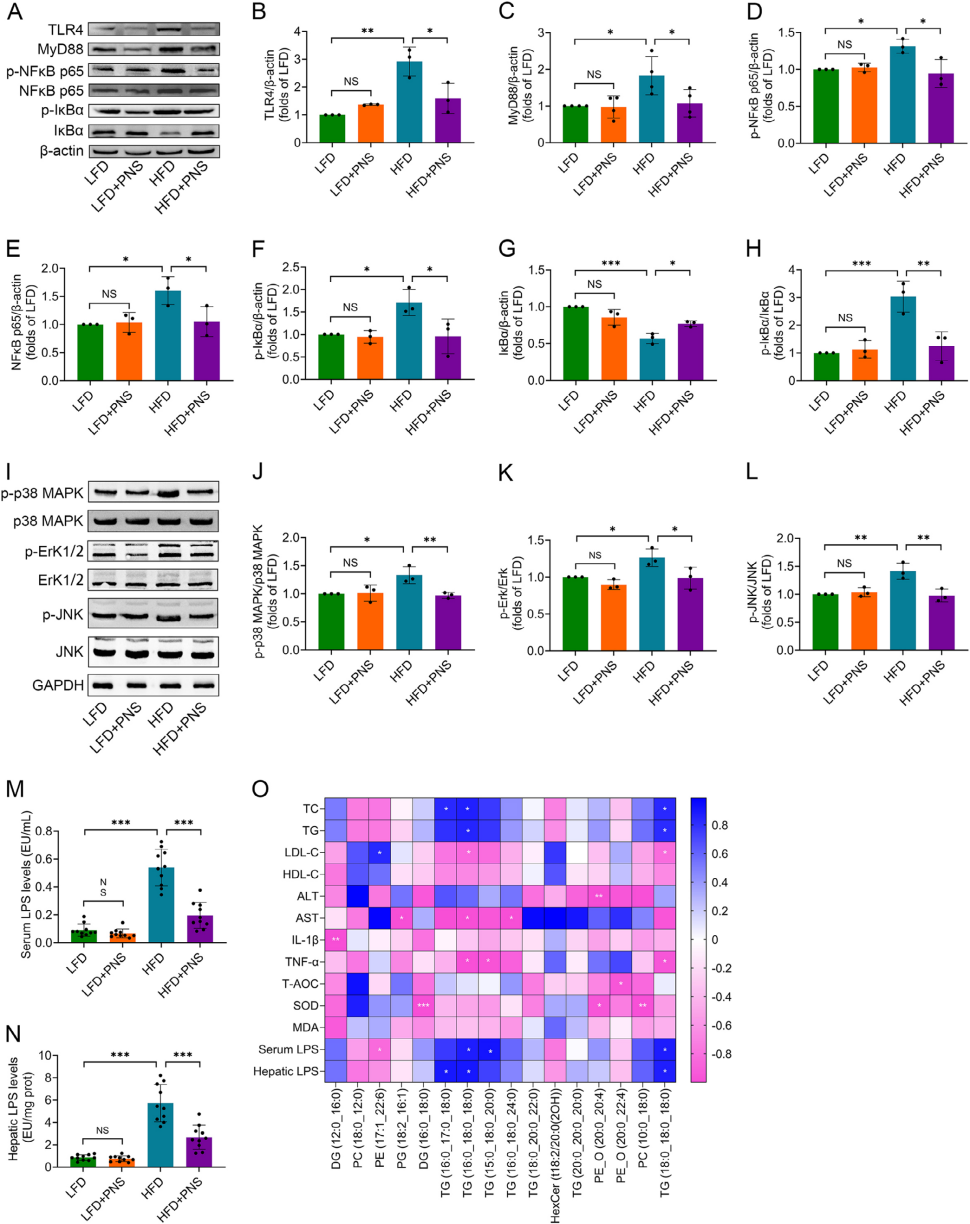
PNS suppress hepatic TLR4/MyD88‐mediated NF‐κB and MAPK signaling in HFD‐fed mice. (A) Representative western blot images of TLR4, MyD88, phospho‐NFκB p65, total NFκB p65, phospho‐IκBα and total IκBα. (B–H) Quantitative analysis of hepatic TLR4 (B; *n* = 3), MyD88 (C; *n* = 4), phospho‐NFκB p65 (D; *n* = 3), NFκB p65 (E; *n* = 3), phospho‐IκBα (F; *n* = 3), IκBα (G; *n* = 3), and the phospho‐IκBα/IκBα ratio (H). β‐Actin served as the loading control for A‐H. (I) Representative western blot images of phospho‐p38 MAPK, total p38 MAPK, phospho‐Erk1/2, total Erk1/2, phospho‐JNK and total JNK proteins. (J–L) Quantitative analysis of the phospho‐p38 MAPK/p38 MAPK ratio (J), phospho‐Erk1/2/Erk1/2 ratio (K), and phospho‐JNK/JNK ratio (L). GAPDH served as the loading control for I‐L. (M, N) LPS levels in serum (M) and liver tissue (N) were measured by ELISA (*n* = 10). (O) A correlation heatmap visualizing Pearson's correlation coefficients between differentially altered hepatic lipids and key metabolic, inflammatory, and oxidative stress parameters in HFD‐fed mice treated with PNS. Data were assessed by one‐way ANOVA followed by Tukey's multiple comparisons test. *p < 0.05, **p < 0.01 and ***p < 0.001; NS, not significant.

Given the well‐established role of TLR4/MyD88‐mediated NF‐κB and MAPK signaling in dysregulating glucolipid metabolism during MASLD progression (Huang et al. 2024), we investigated its modulation by PNS. Compared with LFD controls, HFD‐fed mice showed significantly elevated protein expression of hepatic TLR4 and MyD88, which was effectively reversed by PNS supplementation (Figure 8A–C). Consistent with these upstream changes, PNS treatment also markedly attenuated the HFD‐induced upregulation and phosphorylation of NF‐κB p65 (Figure 8D,E). We further assessed the NF‐κB inhibitory protein IκBα and its phosphorylation status. PNS supplementation counteracted the HFD‐mediated reduction in IκBα expression and increase in IκBα phosphorylation (Figure 8F,G), consequently normalizing the phospho‐IκBα/IκBα ratio (Figure 8H). Furthermore, PNS significantly reversed the HFD‐induced phosphorylation of key MAPK components, including p38, ERK, and JNK (Figure 8I–L). Mechanistically, LPS, a key endogenous TLR4 agonist and inducer of hepatic inflammation (Xu et al. 2023), was elevated in both the serum and liver of HFD‐fed mice. PNS administration significantly reduced LPS levels in these tissues (Figure 8M,N). Importantly, PNS did not significantly alter these pathway components in LFD‐fed mice, indicating a diet‐dependent modulatory effect. Together, these results demonstrate that the alleviation of HFD‐induced liver injury by the 12‐week PNS intervention involves the downregulation of the LPS‐driven TLR4/MyD88‐mediated NF‐κB and MAPK signaling axis.

Spearman correlation analysis was performed to delineate the relationships between significantly altered hepatic lipids and key metabolic, inflammatory, and oxidative stress parameters in HFD‐fed mice treated with PNS. As summarized in Figure 8O, several key patterns emerged. Serum atherogenic lipids, including TC and LDL‐C, showed distinct associations with specific hepatic triglycerides (TGs). Serum TC was positively correlated with hepatic TG (16:0_17:0_18:0), TG (16:0_18:0_18:0), and TG (18:0_18:0_18:0), whereas LDL‐C was positively correlated with PE (17:1_22:6) but negatively correlated with TG (16:0_18:0_18:0) and TG (18:0_18:0_18:0). Furthermore, serum LPS levels exhibited a positive correlation with several TGs [TG (16:0_18:0_18:0), TG (15:0_18:0_20:0), TG (18:0_18:0_18:0)] but a negative correlation with PE (17:1_22:6). Hepatic LPS was positively correlated with TG (16:0_17:0_18:0), TG (16:0_18:0_18:0), and TG (18:0_18:0_18:0). Regarding inflammatory and injury markers, hepatic levels of IL‐1β and TNF‐α were negatively correlated with DG (12:0_16:0) and several TGs, respectively. Serum liver injury markers ALT and AST were negatively correlated with PE_O (20:0_20:4) and a subset of lipids [PG (18:2_16:1), TG (16:0_18:0_18:0), TG (16:0_18:0_24:0)], respectively. Finally, hepatic SOD activity was negatively correlated with DG (16:0_18:0), PE_O (20:0_20:4), and PC (10:0_18:0).

### 3.5 PNS Ameliorated LPS‐Induced Injury in HepG2 Cells by Suppressing TLR4/MyD88 Signaling

**FIGURE 9:**
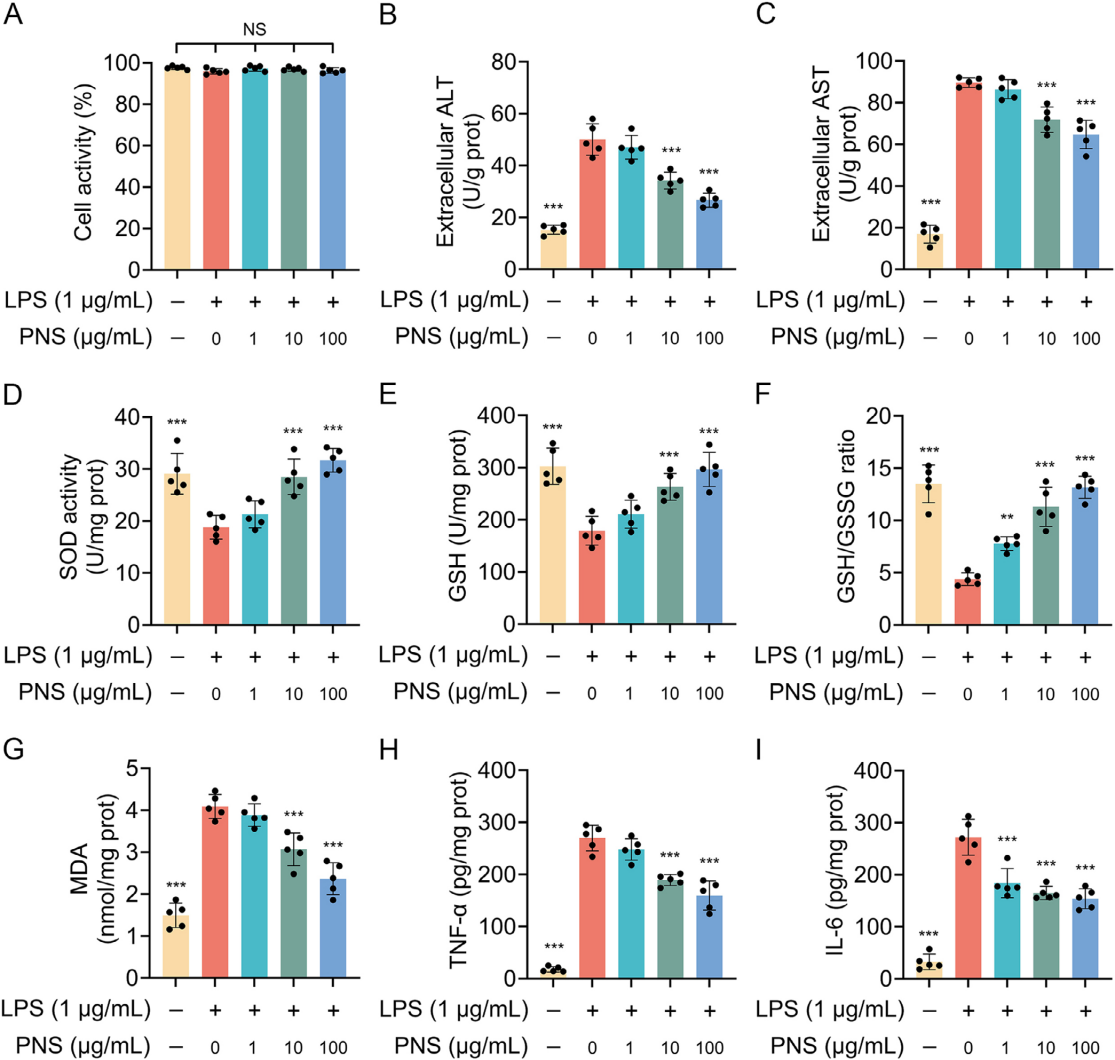
PNS ameliorates LPS‐induced injury in HepG2 cells. (A) Cell viability after treatment with LPS (1 μg/mL) and/or PNS (1, 10, 100 μg/mL) for 48 h (24 h pre‐incubation with PNS followed by 24 h co‐treatment with LPS). (B, C) Levels of ALT (B) and AST (C) in the culture supernatant. (D–G) Oxidative stress parameters: SOD activity (D), GSH level (E), GSH/GSSG ratio (F), and MDA content (G). (H, I) Levels of the pro‐inflammatory cytokines TNF‐α (H) and IL‐6 (I) in the culture supernatant. Data were assessed by one‐way ANOVA followed by Dunnett's test (*n* = 5). **p < 0.01 and ***p < 0.001 versus the LPS alone group; NS, not significant.

To further validate the mechanism observed in vivo, we investigated the effect of PNS in LPS‐stimulated HepG2 cells. Cell viability assays confirmed that neither LPS (1 μg/mL) nor PNS (1, 10, 100 μg/mL) exhibited significant cytotoxicity after the full 48‐h treatment period (24‐h PNS pre‐incubation followed by 24‐h LPS challenge) (Figure 9A). PNS pre‐treatment dose‐dependently attenuated LPS‐induced hepatocyte injury, as evidenced by significantly reduced levels of ALT and AST in the culture supernatant (Figure 9B,C). Furthermore, PNS alleviated LPS‐induced oxidative stress by increasing SOD activity and GSH levels, elevating the GSH/GSSG ratio, and decreasing MDA production (Figure 9D–G). The release of pro‐inflammatory cytokines TNF‐α and IL‐6 was also significantly suppressed by PNS in a dose‐dependent manner (Figure 9H,I).

**FIGURE 10:**
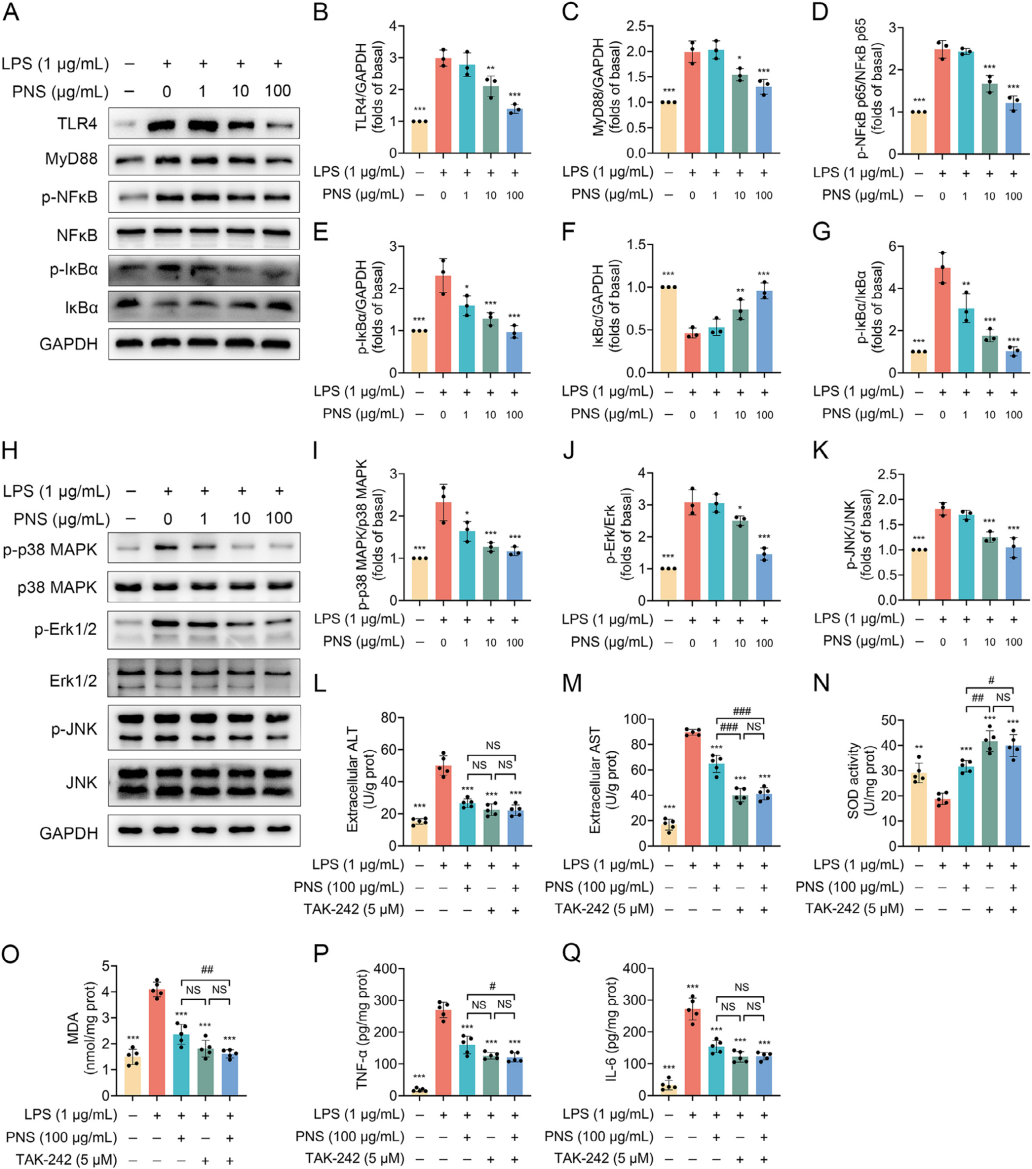
PNS suppress TLR4/MyD88‐mediated signaling in LPS‐stimulated HepG2 cells. (A) Representative western blot images of TLR4, MyD88, phospho‐NFκB p65, total NFκB p65, phospho‐IκBα and total IκBα. (B–G) Quantitative analysis of hepatic TLR4 (B), MyD88 (C), phospho‐NFκB p65/NFκB p65 ratio (D), phospho‐IκBα (E), IκBα (F), and the phospho‐IκBα/IκBα ratio (G). (H) Representative western blot images of phospho‐p38 MAPK, total p38 MAPK, phospho‐Erk1/2, total Erk1/2, phospho‐JNK and total JNK. (I–K) Quantitative analysis of the phospho‐p38 MAPK/p38 MAPK ratio (I), phospho‐Erk1/2/Erk1/2 ratio (J), and phospho‐JNK/JNK ratio (K). GAPDH served as the loading control. (L–Q) Effects of the TLR4 inhibitor TAK‐242 (5 μM) and PNS (100 μg/mL), alone or in combination, on LPS‐induced alterations: ALT (L), AST (M), SOD activity (N), MDA content (O), TNF‐α (P), and IL‐6 (Q) levels. Data were assessed by one‐way ANOVA followed by Dunnett's test (B–K) or Tukey's multiple comparisons test (L–Q) (*n* = 5). *p < 0.05, **p < 0.01 and ***p < 0.001 versus the LPS alone group; ^#^ *p* < 0.05, ^##^ *p* < 0.01 and ^###^ *p* < 0.001; NS, not significant.

Mechanistically, PNS downregulated the LPS‐induced protein expression of TLR4 and MyD88 (Figure 10A–C). Consistent with the in vivo findings, PNS inhibited the activation of the downstream NF‐κB pathway, reducing the phosphorylation of NF‐κB p65 and IκBα while preventing IκBα degradation (Figure 10D–G). Additionally, PNS suppressed the LPS‐induced phosphorylation of key MAPK family members, including p38, ERK, and JNK (Figure 10H–K). As a critical pharmacological validation, the specific TLR4 inhibitor TAK‐242 (5 μM) similarly reversed the LPS‐induced alterations in ALT, AST, SOD, MDA, TNF‐α, and IL‐6 (Figure 10L–Q). Notably, co‐treatment with PNS (100 μg/mL) and TAK‐242 (5 μM) did not yield an additive or synergistic protective effect compared to either treatment alone (Figure 10L–Q), suggesting a functional convergence on the TLR4 signaling axis.

## 4 Discussion

Long‐term HFD consumption is a major contributor to dyslipidemia and hepatic lipid accumulation, serving as a key driver in the pathogenesis of MASLD. PNS, the primary bioactive constituents of *Panax notoginseng*, have been reported to regulate glucose and lipid metabolism (Zhang et al. 2022). In this study, we demonstrate that PNS supplementation effectively attenuates HFD‐induced liver injury in mice, as evidenced by the amelioration of oxidative stress, inflammation, and lipid metabolic dysfunction. Mechanistically, these beneficial effects were associated with the suppression of the hepatic TLR4/MyD88‐mediated NF‐κB and MAPK cascades. Complementary in vitro experiments in LPS‐stimulated HepG2 cells corroborated these findings, showing that PNS downregulate the same pathways and mitigate cellular injury. Notably, the protective effect of PNS converges functionally with that of the specific TLR4 inhibitor TAK‐242, reinforcing the central role of the TLR4 axis in its mechanism of action. Our integrated findings thus reveal that PNS confer hepatoprotection in the context of hyperlipidemia by modulating the TLR4/MyD88‐dependent signaling network.

Systemic lipid abnormalities emerge during the early phases of MASLD and play an active role in its pathogenesis. The link between HFD and hepatic injury has been extensively studied. HFD consumption elevates serum levels of TC, TG, and LDL‐C, while decreasing HDL‐C. These changes are strongly associated with elevated biomarkers of liver damage, including ALT, AST, inflammation, and oxidative stress. HFD‐induced elevation in TC and TG promotes hepatic lipid accumulation, leading to steatosis, inflammatory activation, and increased risk of NAFLD and fibrosis (Li et al. 2016). Furthermore, increased LDL‐C and reduced HDL‐C correlate positively with the severity of liver injury, indicating that dyslipidemia is a significant predictor of hepatic damage (Yang et al. 2025). Dietary interventions and certain bioactive compounds may attenuate HFD‐induced liver injury (Ma et al. 2026). In line with prior studies (Yang et al. 2022; Zhang et al. 2022; Ma et al. 2026), we demonstrated that PNS supplementation significantly improved the circulating lipid profile in HFD‐fed mice, reducing plasma TG, TC, and LDL‐C while increasing HDL‐C. Thus, the beneficial effects of PNS on systemic dyslipidemia likely contribute to the amelioration of hepatic injury by mitigating inflammatory responses and oxidative stress under hyperlipidemic conditions.

Long‐term consumption of HFD leads to significant lipid dysmetabolism linked to MASLD pathogenesis. It has been demonstrated that patients with NAFLD showed alterations in the total serum levels of several lipid classes, including TG, cholesterol esters (CE), lysophosphatidylcholines (LPC), and sphingomyelins (SM) (Flores et al. 2021). However, the role of hepatic lipid dysmetabolism in MASLD progression is less understood. To further elucidate the regulatory mechanisms of hepatic function through PNS supplementation, a nontargeted lipidomic analysis was conducted in liver tissues using UPLC‐TOF/MS.

Our analysis identified a distinct hepatic lipid signature induced by HFD feeding, which was substantially normalized by PNS treatment. Specifically, we observed a marked upregulation of multiple TG species, particularly those enriched with long‐chain saturated fatty acids such as TG (18:0_18:0_18:0), TG (18:0_20:0_22:0), and TG (20:0_20:0_20:0). This pattern is consistent with enhanced de novo lipogenesis and esterification activity, which are key drivers of hepatic steatosis (Ooi et al. 2021). In addition, the elevation of DG (16:0_18:0), a key intermediate in TG synthesis, further supports increased flux through the glycerol‐3‐phosphate pathway (Samuel and Shulman 2018). Concurrent elevation of specific HexCer and ether‐linked phosphatidethanolamine (PE‐O) species suggests concomitant disruptions in sphingolipid and phospholipid metabolism, which have been implicated in insulin resistance and inflammatory activation (Zhu et al. 2022). Conversely, the downregulation of polyunsaturated fatty acid (PUFA)‐containing species, including PE (17:1_22:6) and PG (18:2_16:1), may reflect enhanced lipid peroxidation and a metabolic shift toward more saturated lipid profiles, contributing to membrane instability and lipotoxicity (Cai et al. 2021). The pronounced reversal of these lipidomic alterations by PNS underscores its efficacy in restoring lipid homeostasis, likely via modulation of key transcriptional regulators governing lipogenesis, fatty acid oxidation, and oxidative stress responses (Xu et al. 2021). Furthermore, the observed correlations between specific lipid species and clinical parameters in PNS‐treated mice suggest that lipid pathway modulation constitutes a key mechanism underlying the therapeutic efficacy of PNS. Collectively, these findings position PNS as a multi‐target intervention capable of rectifying specific lipid perturbations that drive HFD‐induced hepatic metabolic dysfunction.

Our results demonstrated that PNS exerted protective effects against HFD‐induced hepatic injury, likely through suppressing the LPS‐driven TLR4/MyD88 signaling axis and its two major downstream branches: the NF‐κB and MAPK pathways. The elevated levels of serum and hepatic LPS in HFD‐fed mice point to endotoxin translocation as a key trigger for TLR4 activation (Hu et al. 2020; Ye et al. 2021). Upon activation, TLR4/MyD88 initiates parallel pro‐inflammatory cascades; NF‐κB regulates the expression of cytokines (e.g., TNF‐α, IL‐6 and IL‐1β) and enzymes involved in oxidative stress (Chen et al. 2024), while the MAPK family members (p38, JNK, ERK1/2) critically control cellular stress responses, apoptosis, additional inflammatory signals, and *de novo* lipogenesis (Davis et al. 2023; Xiao et al. 2024). Our in vitro data in LPS‐stimulated HepG2 cells provide direct validation, showing that PNS concurrently downregulate both the phosphorylation of NF‐κB p65/IκBα and the activation of p38, JNK, and ERK. The functional convergence of PNS with the TLR4 inhibitor TAK‐242, evidenced by the lack of an additive effect, reinforces that its action is integral to this specific signaling axis.

Crosstalk between inflammatory NF‐κB and MAPK signaling allows for synergistic amplification of inflammatory responses and metabolic dysregulation (Guo et al. 2024). The coordinated suppression of both pathways by PNS likely underlies the comprehensive attenuation of hepatic inflammation, oxidative stress (reflected by reduced MDA and elevated SOD/GSH), and lipid metabolic dysfunction observed in vivo. This dual inhibition may restore lipid homeostasis by cooperatively modulating the expression of genes involved in *de novo* lipogenesis, fatty acid oxidation, and cholesterol efflux (Davis et al. 2023; Yang et al. 2023). Therefore, our integrated findings suggest that PNS‐mediated reduction of LPS levels and subsequent downregulation of the TLR4/MyD88‐mediated NF‐κB and MAPK network constitute a central mechanism coordinating its anti‐inflammatory, antioxidant, and lipid‐regulating effects. Future studies employing genetic approaches (e.g., TLR4 knockout) are warranted to establish causal roles and elucidate finer details of the crosstalk between these pathways in PNS‐mediated hepatoprotection.

PNS are characterized by notably low oral bioavailability. For instance, following oral administration of 40 mg/kg PNS in rats, the total bioavailability was reported to be only 1.2%, with maximal plasma concentrations of the major saponins G‐Rg1, G‐Rb1, G‐Rd, and NG‐R1 reaching 6.06, 230, 58.05, and 7.63 ng/mL, respectively (Fu et al. 2023). Despite this poor absorption, PNS still demonstrate significant in vivo pharmacological efficacy, which may be partially attributable to their modulatory effects on gut microbiota (Guo et al. 2020). In addition, there is growing interest in the hepatoprotective roles of individual PNS components (Gong et al. 2022). Given the complex mechanisms through which PNS influence hepatic function and gut microbiota crosstalk, further research is warranted to elucidate the multi‐target mechanisms of specific PNS constituents under hyperlipidemic conditions. Such studies are crucial for evaluating the potential of *Panax notoginseng* in the prevention and treatment of MASLD.

## 5 Conclusions

In summary, this study demonstrates that PNS ameliorates HFD‐induced liver injury by modulating the LPS–TLR4/MyD88 signaling axis and its downstream inflammatory cascades. PNS supplementation significantly attenuated hepatic inflammatory responses and oxidative stress. Mechanistically, these benefits were linked to the suppression of both the NF‐κB and MAPK (p38, JNK, ERK) signaling pathways in vivo, a finding directly corroborated by in vitro experiments in LPS‐stimulated HepG2 cells. The functional convergence of PNS with the specific TLR4 inhibitor TAK‐242 underscores the centrality of the TLR4 pathway in its action. Collectively, our findings provide compelling evidence that PNS supports liver health through the coordinated regulation of lipid metabolism and TLR4‐mediated inflammatory pathways, highlighting its potential as a multi‐targeted dietary supplement for metabolic liver conditions. Further investigation is warranted to explore the translational applicability of PNS and to elucidate the precise crosstalk between the regulated pathways.

## Author Contributions

**Rong Li, Junyu Ma** and **Mengyao Li:** investigation, formal analysis, writing – original draft and writing – review and editing. **Bangzhao Zeng:** methodology, software, writing – review and editing. **Xuexun Li:** methodology, visualization, writing – review and editing. **Xiaoyan Bi** and **Xin Zhao:** software, visualization, writing – review and editing. **Qin Gao:** methodology, writing – review and editing; **Yanling Yao** and **Yang Jiang:** software, writing – review and editing. **Chunmei Zhang:** methodology, visualization, supervision, writing – original draft and writing – review and editing. **Fuli Ya:** funding acquisition, conceptualization, methodology, supervision, writing – original draft and writing – review and editing. All authors contribute to the data curation, validation and approving the final manuscript.

## Funding

This work was supported by Yunnan Fundamental Research Projects (202101AT070033 and 202401AT070081), the National Natural Science Foundation of China (grant No. 82260638), and Yunnan Key Laboratory of Screening and Research on Anti‐pathogenic Plant Resources from Western Yunnan.

## Conflicts of Interest

The authors declare no conflicts of interest.

## Supporting information


**Table S1:** The feed composition for animal diets.

## Data Availability

The data that support the findings of this study are available on request from the corresponding author. The data are not publicly available due to privacy or ethical restrictions.
