# Supplementary material for: Panax notoginseng Saponins Ameliorate High‐Fat Diet‐Induced Liver Injury via Mechanisms Involving TLR4‐Mediated Signaling and Lipid Metabolism
Source: Food Sci Nutr. 2026 Feb 13;14(2):e71544. doi: 10.1002/fsn3.71544 (PMC12903550; doi:10.1002/fsn3.71544)
Supplement: Supplementary file 1 — Table S1: The feed composition for animal diets. [file FSN3-14-e71544-s001.docx]

Supplementary Material

***Panax notoginseng saponins ameliorate high-fat diet-induced liver injury via mechanisms involving TLR4-mediated signaling and lipid metabolism***

Rong Li^1#^, Junyu Ma^1#^, Mengyao Li^1#^, Bangzhao Zeng^1^, Xuexun Li^1^, Xiaoyan Bi^1^, Xin Zhao^1^, Qin Gao^2^, Yanling Yao^3^, Yang Jiang^1^, Chunmei Zhang^4*^, Fuli Ya^1*^

*^1^**Department of Nutrition, School of Public Health,* *Dali University, Dali, Yunnan Province 671000, China;*

*^2^School of Public Health, Jining Medical University, Jining, Shandong Province 272000, PR China;*

### *^3^Department of Nutrition, The Eighth Affiliated Hospital, Sun Yat-sen University, Shenzhen, Guangdong Province 518033, PR China;*

*^4^Department of Laboratory Teaching Center, School of Public Health, Dali University, Dali, Yunnan Province 671000, China;*

^#^ Rong Li, Junyu Ma and Mengyao Li contributed equally to this work.

*Corresponding authors:

Fuli Ya, MD, Ph.D

Department of Nutrition, School of Public Health, Dali University,

No. 22, Wanhua Road, Dali, Yunnan Province, PR China 671000;

E-mail: [yafuli@yeah.net](mailto:yafuli@yeah.net); Tel/Fax: 86-872-2257382

Chunmei Zhang, MD

Department of Laboratory Teaching Center, School of Public Health, Dali University,

No. 22, Wanhua Road, Dali, Yunnan Province, PR China 671000;

E-mail: [ZhangCM2023@yeah.net](mailto:ZhangCM2023@yeah.net)

**1. Supplementary tables, figures and legends**

**Table S1. The feed composition for animal diets.**

| Ingredient | LFD group/  g (kcal) | LFD+PNS group/  g (kcal) | HFD group/  g (kcal) | HFD+PNS group/  g (kcal) |
| --- | --- | --- | --- | --- |
| PNS | 0 | 0.2 (-*) | 0 | 0.2 (-*) |
| Casein | 200 (800) | 200 (800) | 200 (800) | 200 (800) |
| Corn starch | 452.2 (1808.8) | 452.2 (1808.8) | 72.8 (291) | 72.8 (291) |
| Maltodextrin | 75 (300) | 75 (300) | 100 (400) | 100 (400) |
| Sucrose | 172.8 (691.2) | 172.8 (691.2) | 172.8 (691) | 172.8 (691) |
| Soybean oil | 25 (225) | 25 (225) | 25 (225) | 25 (225) |
| Cellulose | 50 (0) | 50 (0) | 50 (0) | 50 (0) |
| Mineral mix | 45 (0) | 45 (0) | 45 (0) | 45 (0) |
| Vitamin mix | 10 (40) | 10 (40) | 10 (40) | 10 (40) |
| L-cystine | 3 (12) | 3 (12) | 3 (12) | 3 (12) |
| Choline bitartrate | 2 (0) | 2 (0) | 2 (0) | 2 (0) |
| Lard | 20 (180) | 20 (180) | 177.5 (1598) | 177.5 (1598) |
| Total | 1050 (4057) | 1050.6 (-*) | 858.1 (4057) | 858.7 (-*) |

* The calories were not available.
